# Effects of Butorphanol With Alfaxalone or Dexmedetomidine on Feline Splenic Size and Appearance on Ultrasound and Computed Tomography

**DOI:** 10.3389/fvets.2021.572146

**Published:** 2021-06-02

**Authors:** Cyrielle Finck, Paulo Steagall, Guy Beauchamp

**Affiliations:** ^1^Department of Clinical Sciences, Faculty of Veterinary Medicine, Université de Montréal, Saint-Hyacinthe, QC, Canada; ^2^Faculty of Veterinary Medicine, Université de Montréal, Saint-Hyacinthe, QC, Canada

**Keywords:** alfaxalone, dexmedetomidine, butorphanol, ultrasound, computed tomgraphy, spleen, feline

## Abstract

The purpose of the study was to determine the effects of intramuscular butorphanol with dexmedetomidine or alfaxalone on feline splenic size, echogenicity, and attenuation using ultrasound and computed tomography (CT). Ten healthy research cats underwent ultrasound and CT without sedation (controls), 15 min after protocol AB (alfaxalone 2 mg/kg and butorphanol 0.2 mg/kg) and 10 min after protocol DB (dexmedetomidine 7 μg/kg and butorphanol 0.2 mg/kg), with a one-week wash-out period between each sedation, using a cross-over study design. Images were randomized and anonymized for evaluation by a board-certified radiologist. On ultrasound, the sedative protocols affected splenic thickness, at the body and the tail (*p* = 0.002 and 0.0003, respectively). *Post-hoc* tests revealed that mean ± SEM thickness was greater after AB (body: 10.24 ± 0.30 mm; tail: 7.96 ± 0.33 mm) than for the control group (body: 8.71 ± 0.30 mm; tail: 6.78 ± 0.33 mm), while no significant difference was observed following DB. Splenic echogenicity was unchanged between treatments (*p* = 0.55). On CT, mean ± SEM splenic volume was increased after AB (37.82 ± 1.91 mL) compared to the control group (20.06 ± 1.91 mL) (*p* < 0.0001), but not after DB (24.04 ± 1.91 mL). Mean splenic attenuation increased after AB (*p* = 0.0009), but not DB. Protocol DB may be preferable for profound sedation in cats while avoiding changes in feline splenic imaging. When protocol AB is selected, splenomegaly should be expected, though mild on ultrasound. The increased splenic attenuation after AB is unlikely to be clinically relevant.

## Introduction

Veterinary patients often require sedation during ultrasonography and other types of imaging examinations. Drug-induced splenomegaly is a source of bias, potentially mimicking pathologies on imaging. This phenomenon has been largely reported in dogs with various drugs (1, 2). For example, splenomegaly caused by the administration of barbiturates and phenothiazine derivatives, such as acepromazine, is well reported in the dog (3–5). Propofol has produced different findings in dogs. One study reported no splenic enlargement on ultrasound (3), whereas another found that splenic volume measured on CT was increased (1). Alfaxalone has also been reported to increase canine splenic volume on CT (2). Conversely, the administration of xylazine (an α_2_-adrenergic receptor agonist) did not lead to an increased splenic size on ultrasound (4), and splenic volume did not significantly differ from controls on CT when using hydromorphone or dexmedetomidine (1). Acepromazine has also been reported to increase splenic echogenicity in the dog (3). The proposed cause of increased attenuation was pooling of the red blood cells into the spleen.

Fewer data exist in the cat when compared with the dog (6, 7). The anatomical differences between the feline and canine spleen, most notably of which being splenic size and its subsequent capacity for blood storage, may lead to a difference in magnitude of measurable changes (8, 9). Sevoflurane anesthesia has been reported to mildly increase splenic size in healthy blood-donor cats (7). A recent study reported that intravenous administration of acepromazine is associated with splenic enlargement on radiographs and ultrasound in healthy cats (6). The same study also reported that intravenous dexmedetomidine, the combination of midazolam and butorphanol intravenously or the combination of dexmedetomidine, butorphanol, and ketamine intramuscularly did lead to an increase in splenic size on radiographs, albeit not significant on ultrasound (6). Another study focused on parenchymal changes during contrast-enhanced ultrasound of the spleen in anesthetized cats. It showed that the combination of butorphanol and propofol could reduce the time to first appearance of contrast and made the splenic perfusion pattern more heterogeneous (9). Splenic size was, however, not specifically investigated in this study. True splenomegaly is important to distinguish from drug-induced splenic congestion because it can represent numerous regenerative, inflammatory, or neoplastic processes (8, 10–12). Therefore, knowledge of the potential effects of sedatives on feline splenic size, echogenicity, and attenuation is essential for establishing both appropriate differential diagnoses of splenomegaly and recommendations when performing ultrasound or CT.

The purpose of the study was to determine the effects of intramuscular butorphanol in combination with dexmedetomidine or alfaxalone on feline splenic size, echogenicity, and attenuation using ultrasound and CT. The hypotheses were that both protocols would induce splenomegaly on ultrasound and CT, though to a lesser degree for dexmedetomidine and butorphanol, but that they would not alter splenic echogenicity or attenuation.

## Materials and Methods

The study protocol was approved by the local animal care committee, *Comité d'éthique de l'utilisation des animaux* (*Université de Montréal*) (18-Rech-1958). The criteria set out by the International Association of Veterinary Editors guidelines for publication of studies including animal research were met.

### Animals

Ten healthy domestic short-hair adult research cats were prospectively recruited. The population sample included nine intact females and one neutered male, with a mean (SD) body weight of 3.50 kg (0.44) and a mean (SD) age of 2.7 years (0.82). Physical examination findings were normal. Five cats from the group had a normal hematology and biochemistry, completed for a previous study. Baseline ultrasound evaluation performed on all cats before sedative administration was normal. In the 7 days prior to the study, the cats were acclimated daily to the plexiglass box used for CT using positive reinforcement with food.

### Study Design

**Figure 1:**
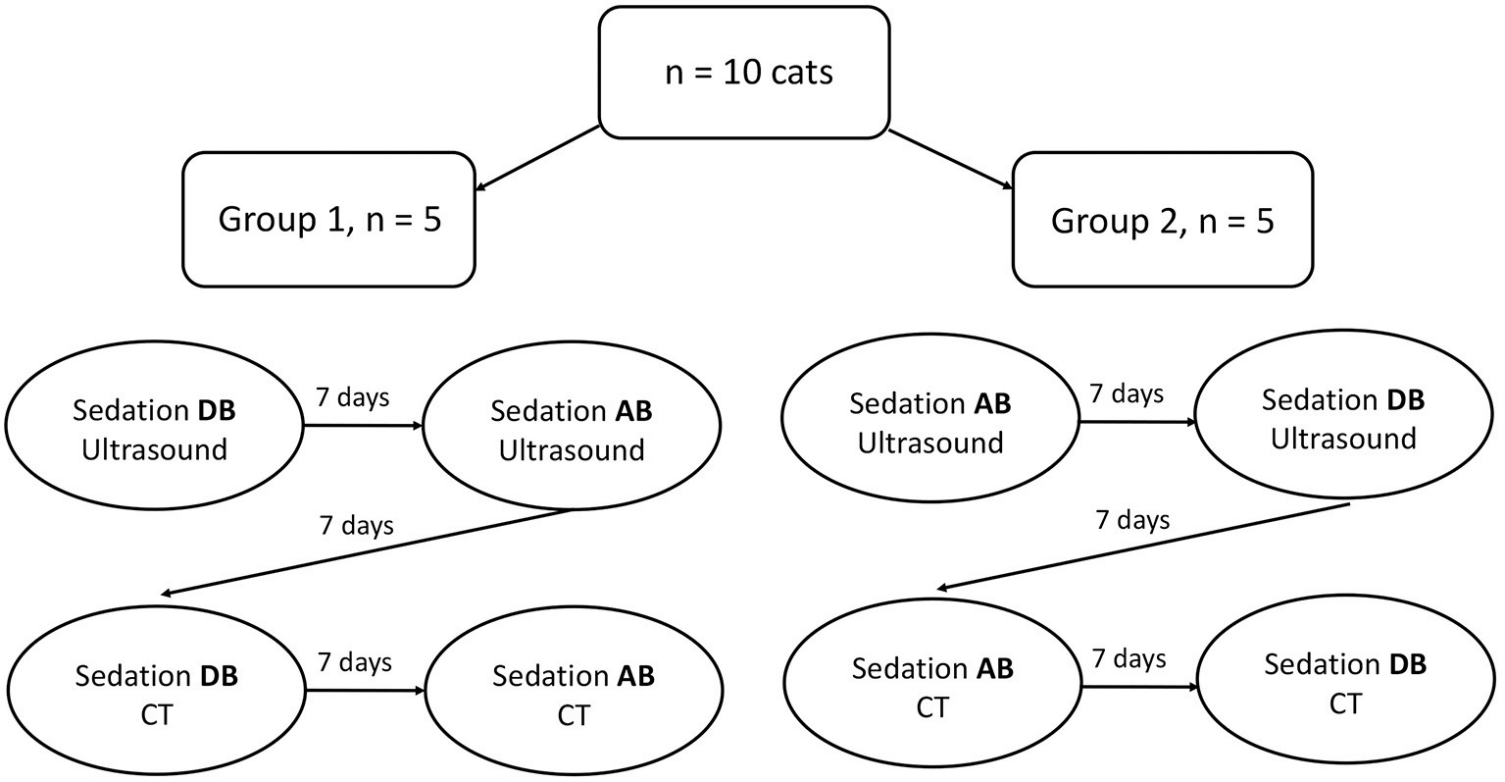
Cross over design for sedatives administration according to groups and imaging tests.

Two sedative protocols were selected for intramuscular administration: AB) alfaxalone (Alfaxan®, Central Sales Limited, Canada) 2 mg/kg with butorphanol (Torbugesic®/MD, Zoetis Canada Inc., Canada) 0.2 mg/kg and DB), and dexmedetomidine (Dexdomitor®, Zoetis Canada Inc., Canada) 7 μg/kg with butorphanol (Torbugesic®/MD, Zoetis Canada Inc., Canada) 0.2 mg/kg. These protocols are often used for sedation in our veterinary teaching hospital and were suggested by a board-certified veterinary anesthesiologist (PS). The two sedative agents of each protocol were combined in the same syringe for simultaneous injection. Intramuscular injections were performed in the lumbar epaxial muscles using a 1 mL syringe and 23-G needle. Prior to each imaging (with or without sedatives), the cats were fasted for 12 h. All cats underwent splenic ultrasound and CT examinations without sedation [controls (C)]. Then, cats were randomly assigned to Group 1 (*n* = 5) or Group 2 (*n* = 5) using a cross-over study design with a 7-day wash-out period between drug administration (Figure 1). Randomization was performed by selecting a number from a bag that corresponded to each cat and allocating the numbers alternatively to Group 1 or 2. Group 1 underwent ultrasound after protocol AB and then DB, followed by CT examination. The order of the sedative protocol was reversed for Group 2 (DB, AB). Cats were placed in a quiet environment before and after sedative administration, in carriers equipped with blankets and in a dimly lit room. Each imaging was initiated 15 min after the administration of protocol AB and 10 min after protocol DB to ensure maximum drug effect. Sedative effects were recorded at the time of imaging according to a scoring system evaluating posture, behavior, and muscle relaxation (13) (Table 1). The three categories were attributed a score that ranged from 0 (least sedative effect) to 3 (maximal sedative effect), and the three values were added together for a total sedative score ranging from 0 to 9. Cats were monitored after sedative administration until the effects subsided, and animals were able to drink and eat.

**Table 1:** Sedative score system.

**Posture score**	
**0**	Able to walk normally
**1**	Moderate ataxia, able to stand up and walk
**2**	Lateral or sternal recumbency but stands up under stimulation
**3**	Lateral recumbency, not able to stand up
**Behavior**	
**0**	Normal response to stimuli (hand-clap and mechanical stimulus at the medial canthus)
**1**	Slower response to stimuli than normal
**2**	Minimal response to stimuli
**3**	No response to stimuli
**Muscle relaxation**	
**0**	Normal jaw and leg tone (resistance to opening of the mouth and flexion of the limbs)
**1**	Mild relaxation of jaw and leg tone
**2**	Moderate relaxation of jaw and leg tone
**3**	Profound relaxation of jaw and leg tone, no resistance

### Imaging

**Figure 2:**
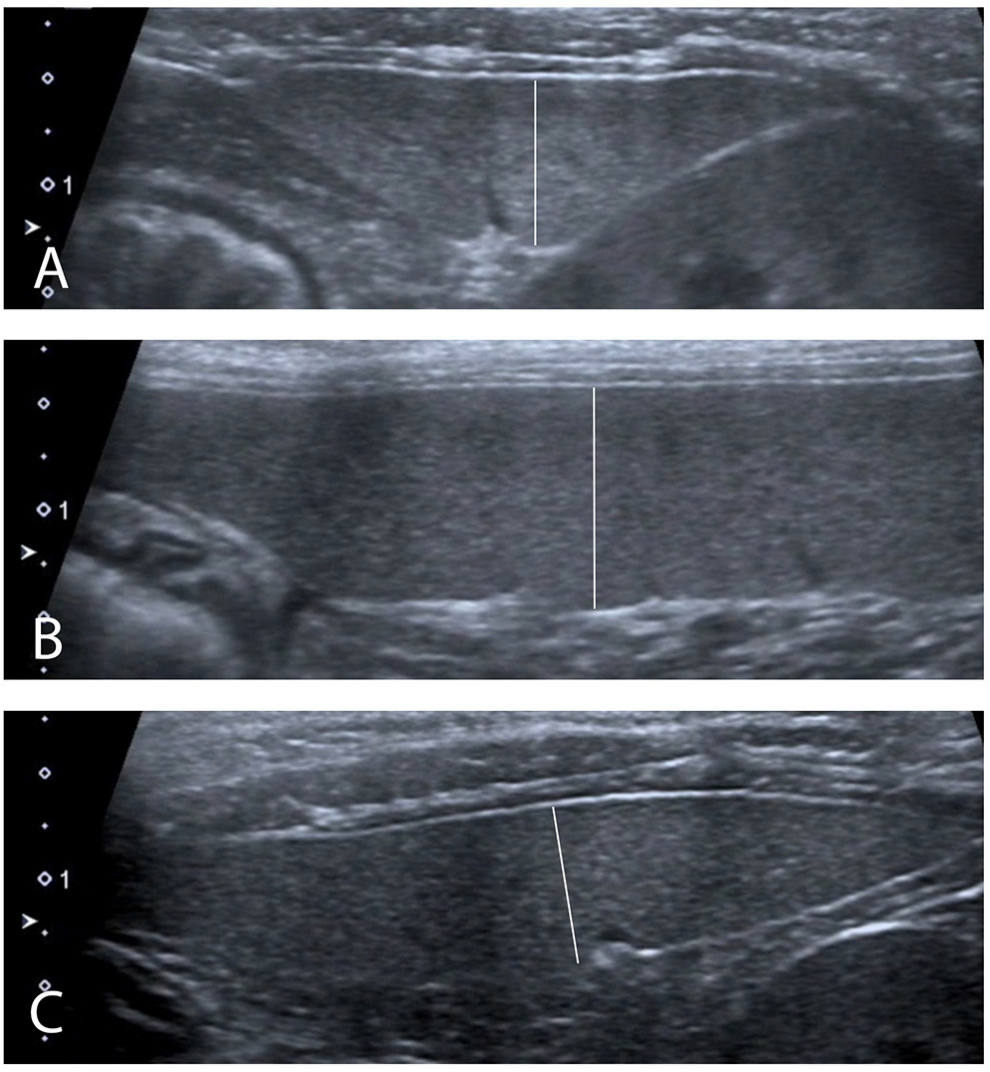
Ultrasound measurements performed **(A)** In transverse plane at the level of the head of the spleen. **(B)** In sagittal plane at the level of the body of the spleen. **(C)** In transverse plane at the level of the tail of the spleen.

Ultrasound images obtained with a 13–18 MHz linear transducer were captured by a radiologist blinded to the sedative protocol used. Depth was set at 3 cm and a single focal zone was positioned at the level of the mesenteric border of the spleen. Gain was adjusted to optimize the image. The entire spleen was scanned in longitudinal and transverse planes. Transverse and sagittal cineloops of the entire spleen were captured. Splenic images were recorded for thickness measurement at three sites, as previously described: head in a transverse plane, body in a sagittal plane, and tail in a transverse plane (Figure 2) (14). For transverse measurements of the head and tail, one caliper was positioned at the mesenteric border of the spleen, adjacent to the indentation created by the splenic vein radicle, and the opposite cursor was placed on the anti-mesenteric border of the spleen, perpendicular to the splenic long-axis. For sagittal measurements of the body, one caliper was placed on the mesenteric border of the spleen, adjacent to the indentation of one vein radicle, and the opposite cursor placed on the anti-mesenteric border, perpendicular to the long-axis of the organ. Splenic echogenicity was recorded using a semiquantitative score determined by comparing its parenchymal appearance to the adjacent liver and left renal cortex on split-screen images: the most echogenic organ was attributed a score of 1, the organ of intermediate echogenicity a score of 2, and the least echogenic organ a score of 3. If two organs had a similar echogenicity, then a score of 1.5 or 2.5 was attributed to both, and a score of 3 or 1 was attributed to the remaining organ, for a total sum of scores between the spleen, kidney, and liver equal to 6. Any focal or multifocal splenic lesion was recorded as absent or present.

**Figure 3:**
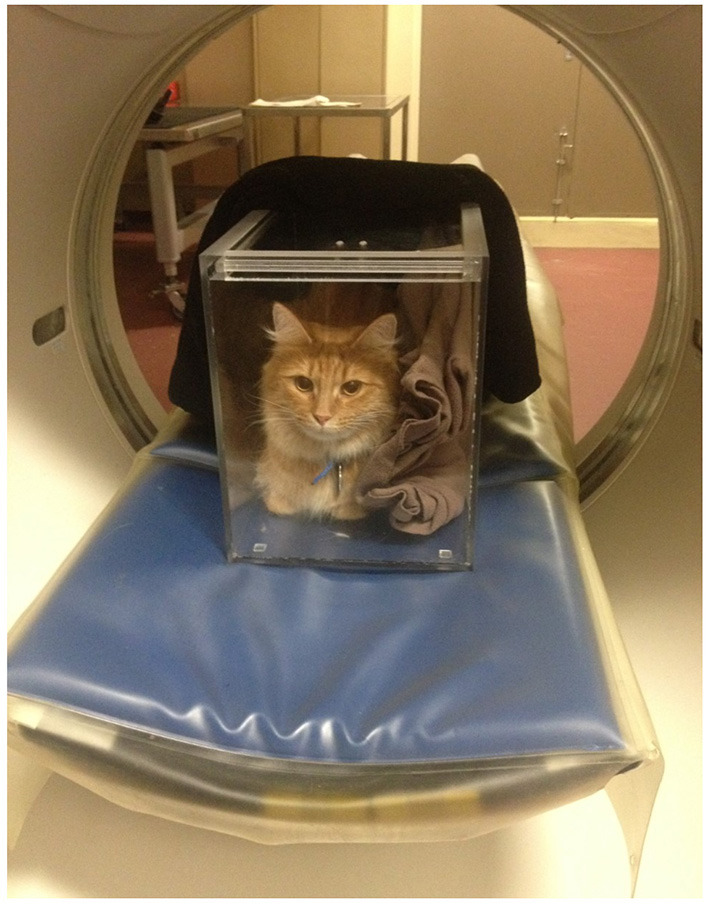
A conscious cat is positioned in sternal recumbency in a plexiglass chamber for an abdominal CT scan. Blankets are placed in the box to act as wedges so that the patient is less tempted to move. The top sliding lid of the box is left ajar to ensure appropriate air flow during the examination.

**Figure 4:**
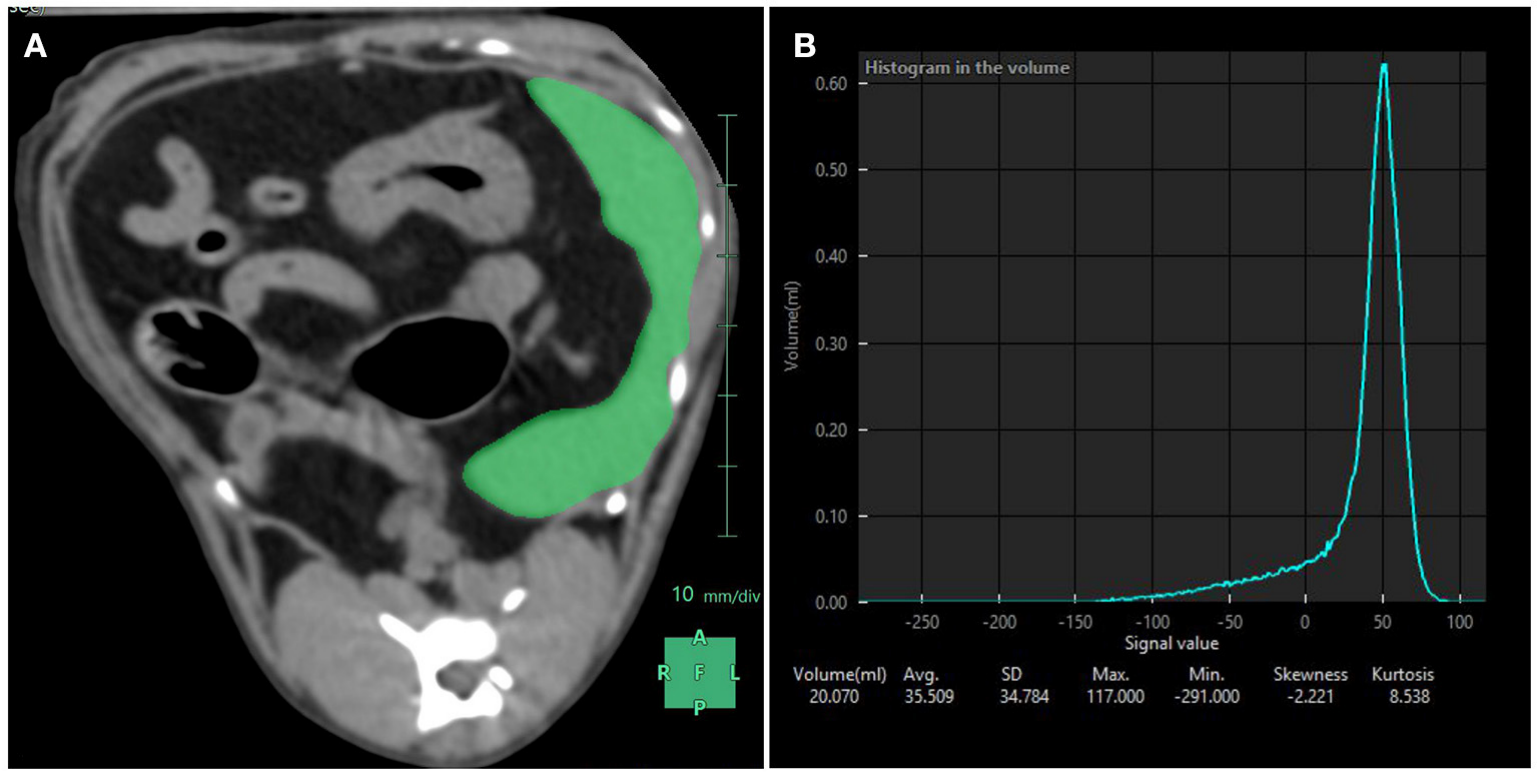
Splenic volume measurements performed with CT. **(A)** Using a dedicated software, the spleen was manually contoured and subsequent region of interests averaged to compute a mask (in green) over the entire splenic volume. **(B)** Resulting splenic attenuation data are depicted as a histogram. At the bottom of the histogram are the splenic volume in mL, the average attenuation in Hounsfield Units (here 35.509 HU) and the corresponding standard deviation, maximum and minimum attenuation values included in the mask, skewness and kurtosis.

Abdominal non-contrast CT studies were obtained from a multi-slice CT scanner and performed with cats placed in sternal recumbency in a plexiglass box (Figure 3). The lid of the box was left ajar to provide air circulation. Images were obtained at 120 kVp, 300 mAs, slice thickness of 1.25 mm, collimator pitch of 0.938:1, rotation tube of 18.75 mm/rot, with a field of view including the abdomen, and reconstructed using of a low-pass filter algorithm. Splenic volume and mean splenic attenuation on CT were calculated using a dedicated viewing software (Synapse 3D, Fudjifilm Medical Systems, USA, Inc.) (Figure 4). All the ultrasound and CT images were randomized and anonymized before analysis for blinded evaluation by a single board-certified radiologist (CF). Randomization was performed with a computed-generated randomization system.

### Statistical Analysis

The sample size calculation for this study was based on data available at the time of study conception. In particular, the smallest splenic size variation after the administration of sedatives in the dog or splenic pathology in the cat was 20% (1, 10). Ten cats would be required to detect such a difference with a power of 0.80 and an alpha level of 0.05.

A linear mixed model was used to compare splenic thickness on ultrasound across the three protocols (C, AB, and DB) at each site (head, body, and tail). In each model, treatment was the fixed effect, and the subject was the random effect as each subject experienced the three treatments. This was followed by Tukey's *post-hoc* tests to determine differences between pairs of means. Splenic echogenicity scores using an ordinal scale were compared across the three protocols with a Cochran–Mantel–Haenszel test for repeated measures. A linear mixed model with protocol as the fixed effect and subject as the random effect was used to compare splenic volumes on CT across the three protocols (C, AB, and AD) followed by Tukey's *post-hoc* tests if necessary. The same approach was used for splenic attenuation. Sedative scores were compared between protocols AB and DB for both imaging modalities (ultrasound or CT) using a Cochran–Mantel–Haenszel test for repeated measures and ordinal scores. All analyses were performed by a statistician and with a statistical software (SAS vs. 9.3, Cary, N.C). Statistical significance was set at *p* < 0.05. Least-squares means ± SEM are shown below unless otherwise stated.

## Results

**Figure 5:**
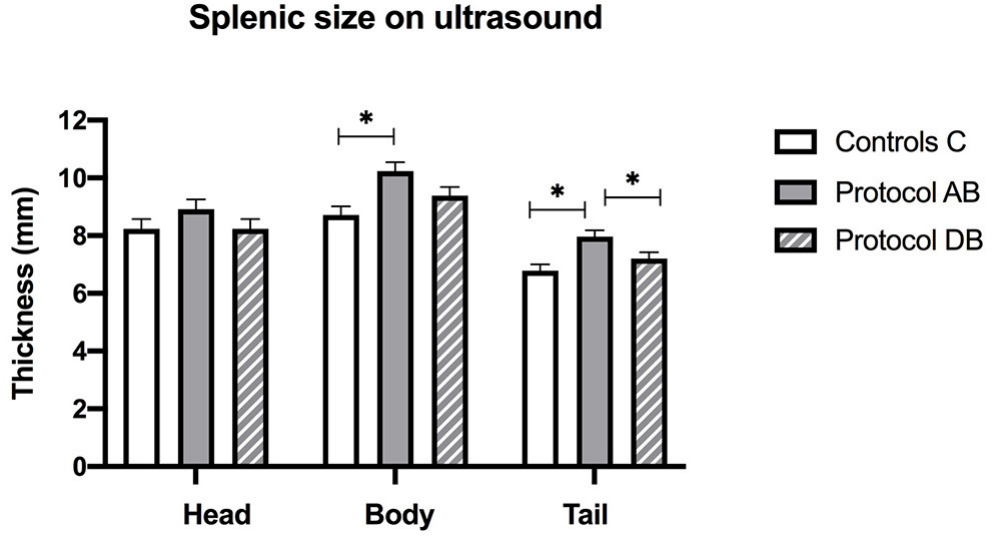
Splenic thicknesses recorded on ultrasound at the three sites (head, body and tail) for the controls and after administration of protocol AB and DB. *Significant differences according to Tukey's *post-hoc* tests (*p* < 0.05). Bars represent the standard error of the mean (SEM).

**Figure 6:**
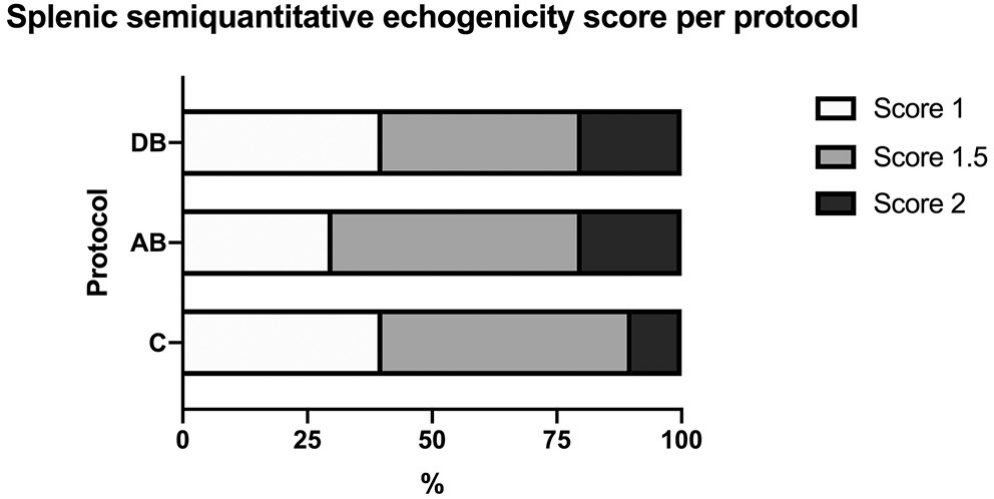
Distribution of splenic semi-quantitative echogenicity scores across the controls C and after the administration of protocols AB and DB.

On ultrasound, an effect of treatment was found for splenic thickness at the body and tail (*p* = 0.002 and 0.0003, respectively). Tukey's *post-hoc* tests indicated that mean splenic thickness at its body increased after AB (10.24 ± 0.30 mm) compared to C (8.71 ± 0.30 mm), but not DB (9.38 ± 0.30 mm) and that mean splenic thickness at the level of the tail was larger in AB (7.96 ± 0.22 mm) than in C (6.78 ± 0.22 mm) or DB (7.21 ± 0.22 mm). Mean splenic thicknesses at the level of the head did not vary significantly among the controls and the two protocols (C: 8.23 ± 0.34 mm; AB: 8.91 ± 0.34 mm; DB: 8.23 ± 0.34 mm) (*p* = 0.24). These results are summarized in Figure 5. Splenic echogenicity scores did not vary significantly across the controls and the two treatment groups (*p* = 0.55) with a median semi-quantitative echogenicity score of 1.5 [range 1–2] for C, 1.5 [range 1–2] after AB and 1.5 [range 1–2] for DB. Figure 6 summarizes splenic scores across the controls and the two treatment groups. No focal splenic lesion was recorded throughout the study.

**Figure 7:**
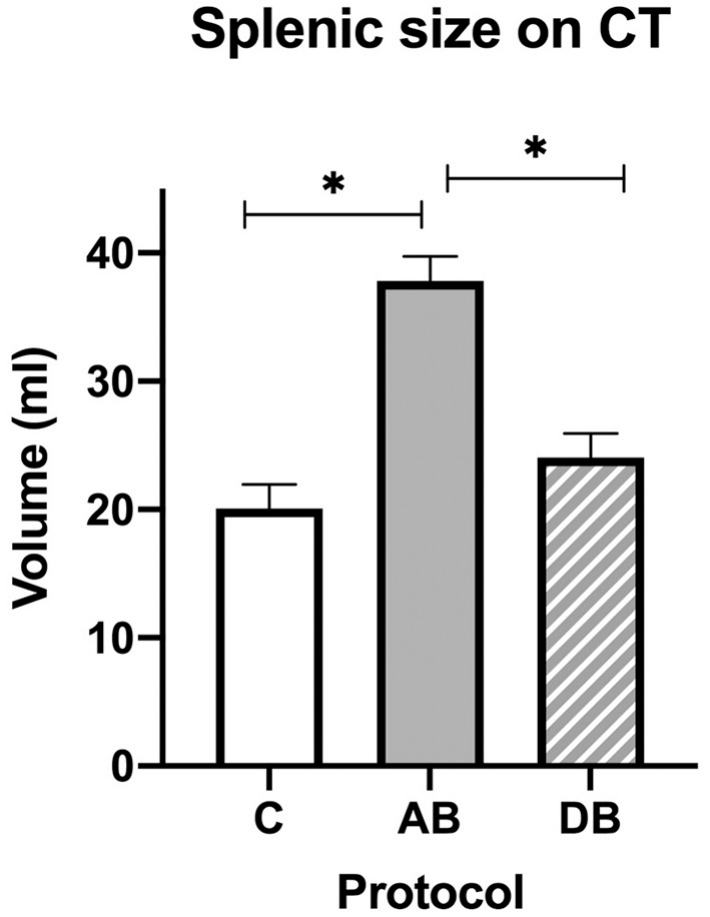
Splenic volumes recorded with CT for the controls and after administration of protocol AB and DB. *Significant differences according to Tukey's *post-hoc* tests (*p* < 0.05). Bars represent the standard error of the mean (SEM).

On CT, an effect of treatment was found for splenic volume (*p* < 0.0001). Tukey's *post-hoc* tests indicated that mean splenic volume significantly increased after administration of AB (37.82 ± 1.91 mL) when compared with C (20.06 ± 1.91 mL), but not after DB (24.04 ± 1.91 mL), and that mean splenic volume was larger after AB than DB (Figure 7). There was also an effect of treatment on splenic attenuation (*p* = 0.0009). The *post-hoc* test revealed that compared to C (32.07 ± 1.83 HU), mean splenic attenuation increased after AB (42.17 ± 1.83 HU) but not after DB (37.72 ± 1.83 HU) and that there was no difference in splenic attenuation between protocols AB and DB.

All cats tolerated restraint in the plexiglass box for the CT examinations, even when without sedation (group C). All non-sedated cats were positioned in sternal recumbency and remained in this position during the CT scan, with little movements, the latter of which were predominant at the level of the head. Successful immobilization of the cats was likely the result of their habituation to the box prior to the study. Although mild respiratory motion was noted in some images, the abdominal studies were subjectively of very good quality.

Median sedative scores with protocol AB were 8.5 [range 3–9] for the ultrasound studies and 9 [range 3–9] for the CT studies. Median sedative scores with protocol DB were 9 [range 7–9] for the ultrasound studies and 9 [range 9–9] for the CT studies. Protocol AB provided a similar sedative effect during ultrasound and CT (*p* = 0.80), as did protocol DB (*p* = 0.32). There was no significant difference in sedative scores between protocol AB and protocol DB during CT (*p* = 0.071) and ultrasound (*p* = 0.071) examinations. All cats remained sedated throughout each imaging session.

## Discussion

The combination of alfaxalone and butorphanol produces a rapid, profound, and short-lasting sedative effect (13, 15). It is preferred by some clinicians at our institution for older patients or those with cardiomyopathies. A previous study showed that cats sedated with this protocol had no significant changes in echocardiographic measurements (13). Our study showed that protocol AB led to a significant increase in splenic volume on CT, and corroborates previously reported data in dogs receiving alfaxalone alone (2). On CT, mean splenic volume increased by 17.76 mL, or an 88.5% increase from its initial volume (controls). Sonographically, changes in thickness were only found at the level of the body and tail and were low in magnitude (mean splenic thickness increased by 1.53 mm at the body, or a 17.5% increase from the controls and by 1.18 mm at the tail, or a 17.4% increase from the controls). This could suggest that the threshold of detection of thickness variation is higher on ultrasound for the head in comparison to other parts of the spleen. Additionally, these numbers are just below the splenic size variation (20%) that was used in the power analysis for the sample size calculation and significantly smaller than the magnitude of volume change assessed with CT, highlighting that thicknesses recorded on the ultrasound images underestimate the total 3-dimensional splenic size increase. An increase in splenic volume with protocol AB is presumed to be mainly attributed to alfaxalone since butorphanol alone does not lead to a significant increase in feline splenic size on ultrasound and radiography (6). In fact, although not statistically significant, there was a trend toward a decrease in feline splenic size following the administration of butorphanol (6). This effect has been shown with other opioids such as hydromorphone. Opioids are thought to have sympatholytic effects, thus increasing vagal tone and causing a reduction in splenic blood flow and size (16–18). Previously proposed mechanisms to explain alfaxalone-induced splenomegaly include a relaxation of the splenic capsule, leading to an increase in its size (1, 2). Blood flow redistribution secondary to a dose-dependent decrease in arterial blood pressure could also contribute to splenomegaly, as reported with propofol (19). Another possible mechanism would be red blood cell sequestration (2).

The intramuscular administration of dexmedetomidine and butorphanol produces rapid and satisfactory onset of sedation in healthy cats and may be used for fractious patients (20–22). Moreover, dexmedetomidine can be antagonized with atipamezole, an α_2_-adrenergic receptors antagonist. This study did not reveal any significant change in splenic appearance on ultrasound or CT after DB. Therefore, this refuted our initial hypothesis, which was theorized after considering recent findings in the cat (6). This previous study described a splenic size augmentation on radiographs after administration of dexmedetomidine or butorphanol used alone in healthy cats, although the trend toward splenic size increase was not significant on ultrasound (6). This difference is likely explained by the fact that two-dimensional radiographic measurements of parts of the spleen are less representative of true splenic size than CT volume calculation of the entire organ used in our study. Radiographic measurements may also be affected by positioning or by inter-observer variability (the latter was high in their study). However, our results corroborate those of another study that showed that dexmedetomidine did not lead to a splenic size increase in dogs assessed with CT when compared to controls (1). The lack of splenic enlargement after dexmedetomidine may be explained by a redistribution of blood flow to vital organs, preventing splenomegaly (23, 24), or to a lack of functional post-synaptic α_2_-adrenoreceptor in the splenic vasculature (25).

The cause of increased splenic attenuation after protocol AB is unclear. It might be due to differential blood cell sequestration. However, the difference was mild, remaining in the Hounsfield Unit range of soft tissue. Therefore, it is unlikely to be clinically relevant, especially considering that relevant splenic parenchymal assessment on CT strongly relies on contrast enhancement pattern. Intravenous contrast medium was not administered in this study as intravenous catheters were not placed.

Sedative effect using AB and DB were adequate in the study without significant differences of the level of sedation between treatments or imaging examination. This is similar to a previous study using similar protocols in blood donor cats (21). All cats, including the controls, tolerated restraint in the plexiglass box for the CT examinations. CT examinations are not routinely performed in non-sedated cats in a plexiglass box or other similar equipment, as the patients would not be acclimated to the device, which could lead to significant motion artifacts. However, abdominal CT studies of heavily sedated patients in a plexiglass chamber are feasible and provide good quality images and may be considered in a clinical setting on a case by case basis. Previous studies have demonstrated the feasibility of thoracic CT imaging in awake cats placed in a plexiglass chamber, with the main pitfall being the risk of motion artifact and repeated scanning (26, 27).

One of the limitations of the study is the lack of comparison between different routes of administration of the sedations (i.e., IM vs. IV). It is also not possible to determine if the observed splenic changes would vary according to dosage or if results would have been different using other combinations (i.e., ketamine) with AB or DB. Yet the protocols used provided a level of sedative effect, which the authors feel would have been optimal to perform imaging studies in a clinical setting. Also, drug protocols were injected simultaneously, thus it is not possible to rule out that pharmacological interactions occurred within the syringe. However, this is current practice for premedication of small animals in clinical practice and drugs were combined immediately before intramuscular administration. A second limitation is that imaging was initiated 15 min after the administration of AB and 10 min after DB, to ensure the maximal sedative effect. Reasonably, all images were collected between the next 5–10 min. Therefore, the effects of the protocols after these timepoints remain unknown. However, other studies show that drug-induced splenomegaly with similar molecules is more significant at 15 min than at 30 min (2) or 2–3 h (6). The time frames used in our study also correspond to median maximal sedative scores recorded for intramuscular administration of alfaxalone in the cat at similar doses (28) and are in line with the reality of a busy clinical practice where ultrasounds are performed shortly after sedation. A third limitation is that splenic echogenicity was not quantified under absolute value. However, in practice, echogenicity assessment is semi-subjective and involves comparison with adjacent structures. To reflect this and to render the study more practical, a semi-quantitative scoring system was thus chosen. Finally, splenic measurements were performed by a single observer. This does not allow to control for any inter-observer variability. However, assessing for inter-observer variability of imaging measurements was not the goal of the study.

## Conclusion

Protocol DB may be preferable to produce profound sedative effect in cats when splenic imaging alterations are to be avoided. Mild splenomegaly should be expected after AB. However, protocol AB is neither likely to explain marked splenomegaly on ultrasound, nor alter splenic echogenicity.

## Data Availability Statement

The original contributions presented in the study are included in the article/supplementary material, further inquiries can be directed to the corresponding author/s.

## Ethics Statement

The animal study was reviewed and approved by Comité d'éthique de l'utilisation des animaux (Université de Montréal).

## Author Contributions

CF, PS, and GB designed the study. PS selected the sedative protocols. CF conducted the study including image analysis and drafted the manuscript. GB performed the statistical analysis. All authors reviewed and approved the final manuscript.

## Conflict of Interest

PS has provided consultancy services and has received speaker honoraria from Zoetis. The remaining authors declare that the research was conducted in the absence of any commercial or financial relationships that could be construed as a potential conflict of interest.
